# Association between anthropometry-based nutritional status and malaria: a systematic review of observational studies

**DOI:** 10.1186/s12936-015-0870-5

**Published:** 2015-09-17

**Authors:** Efrem d’Avila Ferreira, Márcia A. Alexandre, Jorge L. Salinas, André M. de Siqueira, Silvana G. Benzecry, Marcus V. G. de Lacerda, Wuelton M. Monteiro

**Affiliations:** Fundação de Medicina Tropical Dr. Heitor Vieira Dourado, Av. Pedro Teixeira, 25, Dom Pedro, Manaus, AM 69040-000 Brazil; Universidade do Estado do Amazonas, Av. Pedro Teixeira, 25, Dom Pedro, Manaus, AM 69040-000 Brazil; Division of Infectious Diseases, Department of Medicine, School of Medicine, Emory University, 49 Jesse Hill Jr Drive, Atlanta, GA 30303 USA; Instituto Nacional de Infectologia Evandro Chagas, Fundação Oswaldo Cruz, Av. Brasil, 4365, Manguinhos, Rio de Janeiro, RJ 21040-360 Brazil; Instituto de Pesquisas Leônidas and Maria Deane, Fundação Oswaldo Cruz, Rua Terezina, 476, Adrianópolis, Manaus, AM 69057-070 Brazil

**Keywords:** Malaria, *Plasmodium vivax*, *Plasmodium falciparum*, Malnutrition, Anthropometry, Cohort studies

## Abstract

**Background:**

Multiple studies in various parts of the world have analysed the association of nutritional status on malaria using anthropometric measures, but results differ due to the heterogeneity of the study population, species of the parasite, and other factors involved in the host and parasite relationship. The aim of this study was to perform a systematic review on the inter-relationship of nutritional status based on anthropometry and malarial infection.

**Methods:**

Two independent reviewers accessed the MEDLINE and LILACS databases using the same search terms related to malaria and anthropometry. Prospective studies associating anthropometry and malaria (incidence or severity) were selected. References from the included studies and reviews were used to increase the review sensitivity. Data were extracted using a standardized form and the quality of the prospective studies was assessed. Selected articles were grouped based on exposures and outcomes.

**Results:**

The search identified a total of 1688 studies: 1629 from MEDLINE and 59 from LILACS. A total of 23 met the inclusion criteria. Five additional studies were detected by reading the references of the 23 included studies and reviews, totaling 28 studies included. The mean sample size was 662.1 people, ranging from 57 to 5620. The mean follow-up was 365.8 days, ranging from 14 days to 1 year and 9 months, and nine studies did not report the follow-up period. Prospective studies assessing the relationship between malaria and malnutrition were mostly carried out in Africa. Of the 20 studies with malarial outcomes, fifteen had high and five had average quality, with an average score of 80.5 %. Most anthropometric parameters had no association with malaria incidence (47/52; 90.4 %) or parasite density (20/25; 80 %). However, the impact of malnutrition was noted in malaria mortality and severity (7/17; 41.2 %). Regarding the effects of malaria on malnutrition, malaria was associated with very few anthropometric parameters (8/39; 20.6 %).

**Conclusions:**

This systematic review found that most of the evidence associating malaria and malnutrition comes from *P. falciparum* endemic areas, with a significant heterogeneity in studies’ design. Apparently malnutrition has not a great impact on malaria morbidity, but could have a negative impact on malaria mortality and severity. Most studies show no association between malaria and subsequent malnutrition in *P. falciparum* areas. In *Plasmodium vivax* endemic areas, malaria was associated with malnutrition in children. A discussion among experts in the field is needed to standardize future studies to increase external validity and accuracy.

## Background

Nutritional status is a closely tied to immune responses to infection, being on the one hand, an important determinant of the risk and prognosis of infectious diseases, and on the other hand, being directly influenced by infection [1]. This bi-directional pattern of synergistic interaction in which, a worse nutritional status negatively contributes to the development and evolution of infections, whereas infections lead to a worsening of nutritional status, is a crucial phenomenon for the understanding of infections’ population dynamics and to establish control strategies for these diseases [2, 3].

Malaria is a preventable, diagnosable and treatable disease. With recent innovations and roll out of multinational interventions, there are fewer people dying from malaria today than 10 years ago. Nonetheless, malaria is still a deadly disease with approximately 219 million cases and 660,000 deaths in 2013 [4]. Malnutrition is also a major public health problem in tropical areas where malaria prevails, with estimated 38 % stunted, 28 % underweight, and 9 % wasted in Africa [5]. To date, findings from studies evaluating associations between various measures of malnutrition and malaria have been contradictory.

Nutritional status impacts on mortality among children under 5 years due to diarrhoea, respiratory diseases, malaria and measles [6]. In relation to morbidity, a big part of the studies found that children and adolescents with chronic malnutrition (stunting) and low weight for age (underweight) besides thin adult have protection against prevalent cerebral malaria [7–10], stunted and underweight children and adolescents have less prevalence and incidence of hyperparasitaemia [11–16] and, to a lower extent, children and adolescents with wasting or stunting were protected against new episodes of clinical malaria [17, 18]. Although limited by the small number of studies, malnutrition may contribute to deaths from malaria, even though the significance was not high compared with other diseases [2, 7, 18–20]. In contrast, some studies found no association between nutrition and subsequent mortality from malaria [21, 22].

Although several studies in various parts of the world analysed the impact of nutritional status on malaria using anthropometric measures, their results differ due to the heterogeneity of the study population, species of the parasite, and other factors involved in host and parasite relationship. The aim of this study was to perform a systematic review on the evidence for relationship between malaria and subsequent chronic or acute malnutrition based on anthropometry, for the protective effect of malnutrition on malaria outcomes, and for malnutrition increasing the risk of adverse malaria outcomes.

## Methods

### Search strategy

A systematic review was performed in order to identify the available published data on malaria and malnutrition causal interactions. A broad free text search was made using the terms (malaria OR plasmodium) AND [(anthropometry) OR (anthropometric) OR (nutrition)) for PUBMED and (malaria OR plasmodium) AND (anthropometry) OR (anthropometric) OR (nutri$)] for LILACS. Potentially relevant papers in all languages were accessed from MEDLINE (September 2014) and LILACS (September 2014) in order to review full texts. Additional articles were obtained through citation tracking of reviews/opinion articles and original papers. The titles, abstracts, and studies identified in the literature search were assessed by two reviewers. All studies matching the inclusion criteria were reviewed by the authors and disagreement in conclusion was settled through discussion. Articles written in English, Portuguese, Spanish, German and French were included.

### Inclusion criteria, exposures and outcomes

For this study, observational studies in non-pregnant populations and without co-morbidities (co-infections, genetic or metabolic and other chronic diseases) were included. Studies on any of the *Plasmodium* species were included. Only original prospective cohort studies were included, presenting one of the following characteristics of exposure and outcome:

1. Malnutrition assessed by anthropometric measurements as the independent variable and morbidity or mortality from malaria as the outcome.
2. Malaria as an exposure variable and malnutrition assessed by anthropometric measurements as the outcome.

The anthropometric variables gathered in the articles were anthropometric indices [(height/age (H/A), weight/height (W/H), weight/age (W/A), arm circumference/age (AC/A), body mass index (BMI)], measures of skinfold thicknesses, body circumferences and increments in weight, height or anthropometric measures throughout follow-up. As malarial variables, we looked for frequencies of malaria such as incidence or prevalence, parasitaemia, complications and mortality as outcomes. Severity criteria recommended by the World Health Organization (anaemia, cerebral malaria, hypoglycaemia, renal failure, pulmonary oedema, or acute respiratory distress syndrome, circulatory collapse, abnormal bleeding or disseminated intravascular coagulation, haemoglobinuria, seizures, acidosis, hyperbilirubinaemia, hyperpyrexia and prostration) were used [23].

### Data extraction

One independent reviewer (EDF) supervised by a senior reviewer (MAA) extracted the relevant data using a predesigned data extraction form. Disagreements between the two reviewers were resolved by referring to a third reviewer (WMM). Information for article identification, exposure variables and outcome measurement, sample size, age group, level of malaria transmission in the study site, follow-up time, species of *Plasmodium* spp., confounders adjusted in the analysis and type of association were extracted. Data on the type of association (statistical comparison) between the exposure and the outcome were retrieved. The association was classified as neutral when the exposure had no impact on the outcome, as risk when the exposure increased the outcome and as protection when the exposure decreased the outcome.

### Evidence quality evaluation

To assess the quality of the included studies, an adapted questionnaire originated from the *Check List for Measuring Quality* proposed by Downs and Black was applied [24]. The validity of the studies was determined by scores received after the evaluation of 12 questions, according to Table 1. For each question with an affirmative answer, 1 point was assigned to the study. Negative or inconclusive responses did not lead to scoring. Studies with scores >70 % were considered of high quality, while studies with scores 50–69.9 % were considered of intermediate quality score and below 50 % classified as low quality.

**Table 1:** Questions used for evidence quality evaluation in the systematic review

Order	Question
1	The study clearly describes its objectives and hypotheses?
2	The study clearly describes the exposures and outcomes?
3	The study describes basic characteristics of participants (age and gender)?
4	The results of the statistical analysis were presented explicitly (p values and/or confidence intervals)?
5	The study provided estimates of random variability in the results of the main outcome measures (standard deviation, standard error, confidence interval)?
6	The results were adjusted for possible confounding variables through stratification or multivariate analysis?
7	The study informs the loss characteristics (numbers and reasons)?
8	Participants were followed for the same time or the study was adjusted for different follow-up times?
9	Statistical tests used were adequate (non application of parametric statistics for population under 100)?
10	The measures used for the main outcomes were accurate (description of the technique for the diagnosis of malaria and nutritional status)?
11	The demographic characteristics of the groups were comparable or adjusted (age and geographic area)?
12	The participants of different groups were recruited in the same period of time?

## Results

### Selection and general characteristics of the study

**Fig. 1:**
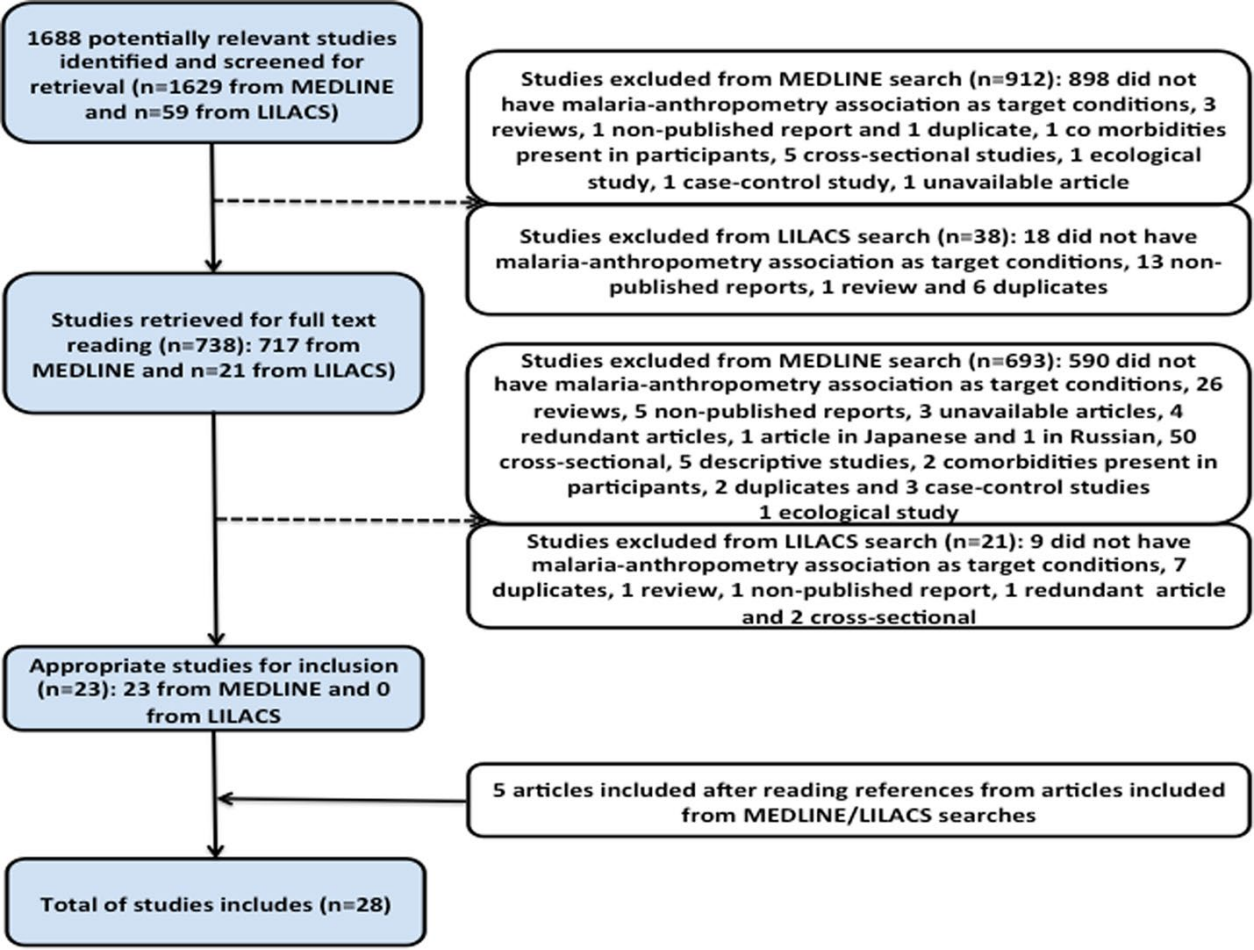
Flow chart of study selection

The search identified a total of 1688 studies: 1629 from MEDLINE and 59 from LILACS. After screening titles and abstracts, 950 publications were excluded due to a lack of information regarding an association between malaria and malnutrition, not being original articles or being duplicates, reviews, reports, case–control, ecological or cross-sectional studies, unavailable articles and presence of co-morbidities in participants (Fig. 1). After reading the remaining 738 full articles, 598 more publications were excluded for similar reasons. A total of 139 studies were taken to a consensus meeting, where 116 did not meet the inclusion criteria. References of the 23 included articles were reviewed, giving 5 extra articles, totaling 28 items for systematic review published from 1977 to 2014.

Twenty-three (82.1 %) studies were conducted in Africa, three (10.7 %) in Oceania (two of Papua New Guinea and Vanuatu), one (3.5 %) in Asia (Vietnam) and one (3.5 %) in South-America (Brazil). African countries with the largest number of studies were Gambia (4), Congo (3) and Ghana (3). Of the 28 included studies, one was done in the 70 s, one in the 80 s, ten in the 90 s, nine in the 2000s and seven in the decade of 2010. Among the 28 included studies, 20 presented anthropometric variables as exposure and had malaria as the outcome, while 13 had malaria as the exposure and malnutrition as the outcome. Five studies conducted evaluations in both directions and were included in both analyses.

Eleven studies did not report the agent responsible for malaria, all from Africa where *P. falciparum* prevails. Fourteen studies had participants with *falciparum* malaria, one with *falciparum* and *vivax*, one with *falciparum*, *malariae* and *vivax* and one with *vivax*/*falciparum* malaria. The setting was characterized as hyperendemic in nine studies, as holoendemic in two, endemic in four, hypoendemic in two, mesoendemic in one and endemicity was not reported in 10 studies. The mean sample size was 662.1 people, ranging from 57 to 5620. The mean follow-up was 365.8 days, ranging from 14 days to 1 year and 9 months, and nine studies did not report the follow-up period. The characteristics and results of these studies are shown in Tables 2 and 3.

**Table 2:** Summary of the results from longitudinal studies with anthropometric measures as exposures and malaria-related outcomes

Identification	Country	Age group/follow-up time	*Plasmodium* species	Sample size	Exposure (undernutrition)	Outcome	Effect measure/intensity	Result	Covariates tested or adjusted
Pazzaglia et al. [28]	Vietnam	Follow-up of 28 days	*P. falciparum*		Grams/day		Multiple regression		Age, race, total duration of infection, duration of primary attack, pre-patent period, incubation period, peak of fever
				58	Increment on weight	Time to peak parasite density	NI (p = 0.050)	Protection	
				42	Increment on weight	Time to peak gametocytaemia	NI (p = 0.050)	Risk	
Snow et al. [29]	Gambia	1 to 4 years (Follow-up of 4 months)	*P. falciparum*		Z-score (mean with SD) (NCHS, 1977)		“t” Test (mean z-score at baseline anthropometry)		None
				138	H/A	Incidence (clinical malaria *vs* no malaria)	−0.96 *vs* −0.89	No association	
				138	W/H	Incidence (clinical malaria *vs* no malaria)	−1.03 *vs* −1.18	No association	
				138	W/A	Incidence (clinical malaria *vs* no malaria)	−1.39 *vs* −1.47	No association	
				34	H/A	Incidence (asymptomatic malaria vs no malaria)	−0.97 *vs* −0.89	No association	
				34	W/H	Incidence (asymptomatic malaria vs no malaria)	−0.96 *vs* −1.18	No association	
				34	W/A	Incidence (asymptomatic malaria vs no malaria)	−1.35 *vs* −1.47	No association	
				59	H/A	High parasite density in clinical malaria (≥5000/µL)	−1.03 *vs* −0.94	No association	
				59	W/H	High parasite density in clinical malaria (≥5000/µL)	−1.32 *vs* −0.91	No association	
				59	W/A	High parasite density in clinical malaria (≥5000/µL)	−1.67 *vs* −1.28	No association	
				15	H/A	High parasite density on asymptomatic infection (≥5000/µL)	−1.84 *vs* −0.81	No association	
				15	W/H	High parasite density on asymptomatic infection (≥5000/µL)	−0.89 *vs* −1.06	No association	
				15	W/A	High parasite density on asymptomatic infection (≥5000/µL)	−1.34 *vs* −1.36	No association	
Van den Broeck et al. [21]	Congo	<5 years	*P. falciparum*		Z-score (<−2) (NCHS, 1977)		RR		None
				64	H/A	Mortality (3 months follow up)	NI (NS)	No association	
				64	W/H	Mortality (3 months follow up)	NI (NS)	No association	
				64	W/A	Mortality (3 months follow up)	NI (NS)	No association	
				64	AC/A	Mortality (3 months follow up)	NI (NS)	No association	
				64	H/A	Mortality (3–30 months follow up)	NI (NS)	No association	
				64	W/H	Mortality (3–30 months follow up)	NI (NS)	No association	
				64	W/A	Mortality (3–30 months follow up)	NI (NS)	No association	
				64	AC/A	Mortality (3–30 months follow up)	NI (NS)	No association	
Genton et al. [22]	Papua New Guinea	11 months to 11 years	*P. falciparum*		Z-score (<−2) (NCHS, 1977)		OR		Age, duration of symptoms, temperature, pulse rate, respiratory rate, parasite density, glycaemia, haemoglobin, maematocri, white cell count
				121	W/A	Mortality (cerebral malaria)	0.98 (0.65–1.49)	No association	
Olumese et al. [7]	Nigeria	1 to 5 years	*P. falciparum*		Percentile ≤80 % (NCHS, 1977)		Fisher’s exact test		None
				57	W/A	Death or recover with neurological deficit on cerebral malaria	NI (p = 0.037)	Risk	
Renaudin et al. [20]	Chad	<5 years	*P. falciparum*		Z-score (<−2) (NCHS, 1977)		X^2^ (Chi-square)		None
				227	W/H	Mortality	NI (NS)	No association	
Williams et al. [30]	Vanuatu	<10 years	*P. falciparum*/*P*. *vivax*		Z-score (<−2) (NCHS, 1977)		RR		None
				702	W/H	Incidence (*P. falciparum*	NI (NS)	No association	
				702	W/A	Incidence (*P. falciparum*	1.1 (0.57–2.1)	No association	
				702	W/H	Incidence (*P.vivax* **)**	NI (NS)	No association	
				702	W/A	Incidence (*P.vivax* **)**	2.6 (1.5–4.4)	Risk	
Man et al. [2]	Gambia	<5 years	*P. falciparum*		Z-score (<−2) (NCHS, 1977)		RR		None
				5,620	W/A	Mortality	NI (S)	Risk	
				1,292	W/A	Cerebral malaria	NI (S)	Risk	
				1,473	W/A	Malarial anaemia	NI (S)	Risk	
Genton et al. [16]	Papua New Guinea	10 months to 10 years	*P. falciparum*/ *vivax*/*malariae*		Z-score (<−2) (NCHS, 1977)		RR		Age, bednet use
				136	H/A	Incidence (*P.* spp.)	1.1 (0.97–1.24)	No association	
				136	W/H	Incidence (*P.* spp.)	0.95 (0.81–1.11)	No association	
				136	H/A	Incidence (*P. falciparum* **)**	1.13 (0.98–1.29)	No association	
				136	W/H	Incidence (*P. falciparum*)	0.92 (0.77–1.11)	No association	
				136	H/A	High parasite density (*P. falciparum*≥5,000/µL)	1.19 (1.01–1.40)	Protection	
				136	W/H	High parasite density (*P. falciparum*≥5,000/µL)	0.96 (0.77–1.19)	No association	
				136	H/A	High parasite density (*P. falciparum*≥10,000/µL)	1.18 (0.98–1.41)	No association	
				136	W/H	High parasite density (*P. falciparum*≥10,000/µL)	0.97 (0.75–1.24)	No association	
				136	H/A	Incidence (*P. vivax*)	0.96 (0.73–1.25)	No association	
				136	W/H	Incidence (*P. vivax*)	0.95 (0.70–1.28)	No association	
				136	H/A	Incidence (*P.malariae*)	0.83 (0.44–1.57)	No association	
				136	W/H	Incidence (*P.malariae*)	0.90 (0.49–1.63)	No association	
Schellenberg et al. [31]	Tanzania	<4 years (Follow-up of 1 year)	*P. falciparum*		Percentile ≤25 (NCHS, 1977)		OR		None
				651	W/A (1-7 months)	Mortality	3.2 (1.2–8.9)	Risk	
				1,620	W/A (8 months–4 years)	Mortality	3 (1.5–6.2)	Risk	
					Percentile >25–50 (NCHS, 1977)				
				651	W/A (1–7 months)	Mortality	2.6 (1–6.9)	No association	
				651	W/A (8 months–4 years)	Mortality	1.9 (0.8–4.2)	No association	
Tonglet et al. [32]	Congo	<2 years (Follow-up of 3 months)	*P. falciparum*	842	Median <25 (NCHS, 1977) <9 months		OR		Age, gender, time of enrolment, malaria during preceding month, diet, adequate growth as judged by care-taker
				842	H/A	Incidence	1.16 (0.54–1.77)	No association	
				842	H/A increment	Incidence	1.53 (0.95–2.11)	No association	
				842	W/A	Incidence	1.31 (0.68–1.94)	No association	
				842	W/A increment	Incidence	1.05 (0.40–1.69)	No association	
				842	AC/A	Incidence	2.32 (1.68–2.97)	Risk	
				842	AC/A increment	Incidence	0.98 (0.35–1.60)	No association	
					Median <25 (NCHS, 1977) ≥9 months				
				842	H/A	Incidence	0.71 (0.28–1.14)	No association	
				842	H/A increment	Incidence	0.74 (0.31–1.16)	No association	
				842	W/A	Incidence	0.68 (0.24–1.11)	No association	
				842	W/A increment	Incidence	0.70 (0.28–1.13)	No association	
				842	AC/A	Incidence	0.97 (0.54–1.40)	No association	
				842	AC/A increment	Incidence	1.10 (0.68–1.51)	No association	
Deen et al. [33]	Gambia	<5 years (Follow-up of 20 weeks)	*P. falciparum*		Z-score (<−2) (NCHS, 1977)		RR		Age, gender, ethnicity
				487	H/A	Incidence	1.35 (1.08–1.69)	Risk	
				487	W/H	Incidence	0.87 (0.69–1.10)	No association	
				487	W/A	Incidence	1.01 (0.82–1.26)	No association	
Muller et al. [34]	Burkina Faso	6 to 30 months (Follow-up of 6 months)	*P. falciparum*		Z-score (≤−2) (NCHS, 1977)		RR		Age, gender, bednet use, socioeconomic status, ethnicity
				685	H/A	Incidence	1 (0.9–1.1)	No association	
				685	W/H	Incidence	1 (0.9–1.1)	No association	
				685	W/A	Incidence	1 (0.9–1.2)	No association	
				685	H/A	High parasite density (≥5,000/µL)	1 (0.9–1.2)	No association	
				685	W/H	High parasite density (≥5,000/µL)	1 (0.9–1.2)	No association	
				685	W/A	High parasite density (≥5,000/µL)	1 (0.9–1.2)	No association	
				685	H/A	High parasite density (≥10,000/µL)	0.8 (0.5–1.4)	No association	
				685	W/H	High parasite density (≥10,000/µL)	0.8 (0.5–1.4)	No association	
				685	W/A	High parasite density (≥10,000/µL)	1 (0.5–1.8)	No association	
Mockenhaupt et al. [35]	Ghana	6 months to 9 years	*P. falciparum*		Z-score (<−2) (NCHS, 1977)		OR		Age, gender, shock, respiratory distress, haemoglobinuria, multiple convulsions, severe anemia, jaundice, prostration, cerebral malaria, impaired consciousness, hyperlactataemia hyperparasitaemia hypoglycaemia, hyperpyrexia
				285	W/H	Mortality (severe malaria)	2.8 (1.1–7.0)	Risk	
Nyakeriga et al. [36]	Kenia	28 to 60 months (Follow-up of 1 year and 5 months)	*P. falciparum*		Z-score (<−2) (NCHS, 1977)	Incidence	RR		Age, ethnicity, season, haemoglobin genotype
				341	H/A	Incidence	1.09 (0.92–1.28)	No association	
				341	W/A	Incidence	0.96 (0.78–1.19)	No association	
Danquah et al. [37]	Ghana	3 months to 2 years (Follow-up of 1 year and 9 months)	*P. falciparum*		Z-score (≤−2) (WHO, 2006)		RR		Season, food availability
				1,200	H/A, L/A, W/H and W/A combined index	Incidence (non-severe malaria)	NI (NS)	No association	
				1,200	H/A, L/A, W/H and W/A combined index	Incidence (assymptomatic malaria)	NI (NS)	No association	
Fillol et al. [17]	Senegal	12 to 70 months (Follow-up of 25 weeks)	*P. falciparum*		Z-score (<−2) (WHO, 2006)		OR		Age, gender, site of residence, presumptive antimalarial treatment
				874	H/A	Incidence	NI (NS)	No association	
				874	W/H	Incidence	0.33 (0.13–0.81)	Protection	
				874	W/A	Incidence	NI (NS)	No association	
				874	H/A	High parasite density (>300 parasites/µL)	2.42 (1.12–5.24)	Risk	
				874	W/H	High parasite density (>300 parasites/µL)	0.48 (0.04–5.34)	No association	
				874	W/A	High parasite density (>300 parasites/µL)	0.55 (0.35–2.66)	No association	
Arinaitwe et al. [38]	Uganda	6 weeks to 1 year (Follow-up of 1 year and 4 months)	*P. falciparum*		Z-score (<−2) (WHO, 2006)		RR		Age, site of residence, chemoprophylaxis, breastfeeding, HIV status
				99	H/A	Incidence	1.24 (1.03–1.48)	Risk	
				99	W/A	Incidence	1.12 (0.86–1.46)	No association	
Mitangala et al. [15]	Congo	6 to 59 months (Follow-up of 1 year)	*P. falciparum* (99 %)/*malariae*/mixed malaria (*P. falciparum* + *P. ovale*)		Z-score (<−3) (WHO, 2006)		OR		Age, season
				790	H/A	Incidence	1.21 (0.76–1.92)	No association	
				790	W/H	Incidence	1.46 (0.84–2.53)	No association	
				790	W/A	Incidence	1.13 (0.82–1.55)	No association	
				790	AC	Incidence	1.14 (0.71–1.83)	No association	
				787	H/A	Incidence (high parasite density -≥5000/µL)	0.48 (0.25–0.91)	Protection	
				787	W/H	Incidence (high parasite density -≥5000/µL)	0.87 (0.38–1.99)	No association	
				787	W/A	Incidence (high parasite density -≥5000/µL)	0.85 (0.53–1.35)	No association	
				787	AC	Incidence (high parasite density -≥5000/µL)	0.83 (0.44–1.55)	No association	
Alexandre et al. [39]	Brazil	1 month to 14 years (Follow-up of 12 months)	*P. vivax* (72.6 %)/*P. falciparum*(22.5 %)/Mixed malaria *P. vivax* + *P. falciparum*(4.9 %)		Z-score (<−2) (WHO, 2006; WHO, 2007)		HR		Age, gender, maternal education, socioeconomic status
				202	H/A	Incidence	0.31 (0.10–0.99)	Protection	

*BMI* body mass index, *H/A* height/age, *W/H* weight/height, *W/A* weight/age, *AC/A* arm circumference/age, *L/A* length/age, *SD* standard-deviation, *NS* non-significant, *NI* non-informed

**Table 3:** Summary of the results from longitudinal studies with malaria patients and nutritional-related outcomes

Identification	Country	**Age group/follow-up time**	*Plasmodium* species	Sample size	Exposure	Outcome	Effect measure/statistic and CI	Result	Covariates tested or adjusted
Rowland et al. [40]	Gambia	6 months to 3 years (Follow-up of 1 year)	*P. falciparum*				Regression		None
				152	Incidence	Increment on weight	1.072 (268) (*p* = <0.001)	Risk	
				152	Incidence	Increment on weight	7.0 (6.0) (NS)	No association	
Williams et al. [30]	Vanuatu	<10 years	*P. falciparum*/*vivax*			Z-score (<−2) (NCHS, 1977)	IRR (Incidence rate ratio)		None
				911	Incidence (*P. falciparum*)	W/H	NI (NS)	No association	
				911	Incidence (*P. falciparum*)	W/A	1.3 (0.9–1.9)	No association	
				911	Incidence (*P. vivax*)	W/H	2.2 (1–4.9)	Risk	
				911	Incidence (*P. vivax*)	W/A	1.3 (0.9–2.0)	No association	
Hautvast et al. [41]	Zambia	6 months to 3 years and 4 months (Follow-up of 1 year and 9 months)	*P. falciparum* (94 %)/Mixed malaria(19 %)		Parasite density (mean with SD)	Z-score (mean with SD)	*r* (Pearson’s)		Age, gender, socioeconomic status, maternal height, haemoglobin, albumin, zinc, retinol, thyrotropin, iron, ferritin, parasite density, C-reative protein, alfa-acid glycoprotein
				108	Cohort 1 Parasite density on period 1 (age range of 6–9 months) *vs Z*-score on period 2 (age range of 14–20 months)	H/A	−0.30 (*p* = 0.005)	Risk	
				102	Cohort 2 Parasite density on period 2 (age range of 14–20 months) *vs Z*-score on period 3 (age range of 22–30 months)	H/A	−0.28 (*p* = 0.03)	Risk	
Deen et al. [33]	Gambia	<5 years (Follow-up of 20 weeks)	*P. falciparum*			Z-score (<−2) (NCHS, 1977)	“t” Test		Age, gender, ethnicity
				392	Incidence	H/A	NI (NS)	No association	
				392	Incidence	W/H	NI (NS)	No association	
				392	Incidence	W/A	NI (NS)	No association	
Friedman et al. [42]	Kenia	12 to 35 years	*P. falciparum*		Parasite density (mean with SD)	Z-score (mean with SD) (CDC/NCHS, 2000)	B (Pearson’s)		Age, parasite density, peripheral blood mononuclear cell production of TNF-alfa in response to mitogen or malarial antigens
				147	Whole cohort Parasite density in the previous transmission period *vs* Z-score and Kg/m^2^	BMI/Age	−0.201 (*p* = 0.11)	No association	
				83	Tanner stage <3 (prepubescent) Parasite density in the previous transmission period *vs* Z-score	Kg/m^**2**^ BMI (mean with SD) <17 kg/m^2^ (≥20 years)	−0.324 (*p* = 0.02)	Risk	
				83	Tanner stage ≥3 (adult) Parasite density in the previous transmission period *vs* Kg/m^2^	BMI (mean with SD) <17 kg/m^2^ (≥20 years)	0.16 (*p *= 0.51)	No association	
Nyakeriga et al. [36]	Kenia	28 to 60 months (Follow-up of 1 year and 5 months)	*P. falciparum*			Z-score (<−2) (NCHS, 1977)	RR	Risk	Age, ethnicity, season, haemoglobin genotype
				340	Incidence	H/A	1.89 (1.01–3.53)	Risk	
				340	Incidence	W/A	1.33 (0.64–2.70)	No association	
Sowumi et al. [43]	Nigeria	<13 years (Follow-up of 14 days)	*P. falciparum*			Fall in weight	OR		Age, gender, fever, duration of illness, parasitaemia, haematocrit, liver and spleen enlargement
				432	Parasite density(≥100,000/µl parasites)	≤5 % from admission to d14	1.21 (0.72–2.01)	No association	
Danquah et al. [37]	Ghana	3 months to 2 years (Follow-up of 1 year and 9 months)	*P. falciparum*			Z-score (≤−2) (WHO, 2006)	GEE (General estimating equation)		Season, food availability
				1200	Incidence	H/A or L/A	−0.18 (*p *= 0.01)	Risk	
				1200	Incidence	W/H	NI (NS)	No association	
				1200	Incidence	W/A	NI (NS)	No association	
Kang et al. [44]	Ghana	3 months to 2 years (Follow-up of 1 year and 9 months)	*P. falciparum*			Z-score (<−2) (WHO, 2006)	RR		Gender, birth weight, birth season, ethnicity group, alpha-thalassaemia, village of birth, mother’s occupation, mother’s education, family’s financial status, mosquito protection, sickle cell trait
				884	Incidence	H/A or L/A	0.32 (0.09–1)	No association	
Olney et al. [45]	Zanzibar	<13 years (Follow-up of 6 months)	*P. falciparum*			Z-score (mean with SD) (WHO, 2006)	Regression		None
				247	Incidence 5–9 months	H/A	0.008 (NS)	No association	
				247	Incidence 10–14 months	H/A	−0.063 (NS)	No association	
Muhangi et al. [46]	Uganda	Birth to 1 year (Follow-up of 1 year)	*P. falciparum*			Z-score (<−2) (WHO, 2006)	OR		Gender, number of living children in the family, early weaning, maternal age, maternal education, socio-economic status, low birth weight, HIV exposure
				1502	Incidence	L/A	2.12 (1.38-3.27)	Risk	
				1502	Incidence	W/L	NI (NS)	No association	
				1502	Incidence	W/A	NI (NS)	No association	
Padonou et al. [47]	Benin	Birth to 18 months (Follow-up of 18 months)	*P. falciparum*			Z-score (WHO, 2006)	Coefficient		Birth place, mother’s age, maternal short stature and low weight status, parity, number of pre-natal visits, marital status, mother’s education, bed net protection, household wealth score, birth weight and length, gender, gestational age, prematurity, intra-uterine growth retardation, maternal anemia, placental malaria infection, use of intermittent preventive treatment
				520	Incidence	H/A	−0.03(0.02) (*p* = 0.15)	No association	
				520	Incidence	W/H	−0.01 (0.02) (*p* = 0.74)	No association	
Alexandre et al. [39]	Brazil	1 month to 14 years (Follow-up of 12 months)	*P. vivax* (72.6 %)/*P. falciparum* (22.5 %)/Mixed malaria *P.vivax* + *P. falciparum* (4.9 %)			Cm/year (WHO, 2006; WHO, 2007)	OR		Age, gender, maternal education, socioeconomic status
				39	Incidence	≤5 years Increment on height	1.1 (0.2–6.4)	No association	
				108	Incidence	5–10 years Increment on height	4.0 (1.4–11.4)	Risk	
				55	Incidence	10–14 years Increment on height	1.1 (0.2–4.8)	No association	
				39	Incidence	Z-score (<−2) ≤5 years H/A	6.9 (0.3–161.6)	No association	
						Z-score (<−2) (WHO, 2006; WHO, 2007) 5–10 years			
				108	Incidence	H/A	0.9 (0.3–3.1)	No association	
				108	Incidence	W/A	5.1 (0.5–45.9)	No association	
				108	Incidence	BMI/A	4.2 (0.4–39.1)	No association	
						Z-score (<−2) (WHO, 2006; WHO, 2007) 10–14 years			
				55	Incidence	H/A	0.4 (0.1–2.2)	No association	
				55	Incidence	BMI/A	1.0 (0.1–17.0)	No association	

*BMI* body mass index, *H/A* height/age, *W/H* weight/height, *W/A* weight/age, *AC/A* arm circumference/age, *L/A* length/age, *SD* standard-deviation, *NS* non-significant

### Malnutrition as exposure for malaria

Twenty studies with malaria-related outcomes were included, twelve evaluating incidence, seven mortality and six parasitaemia. Five studies evaluated more than one type of outcome.

#### Anthropometric parameters

**Fig. 2:**
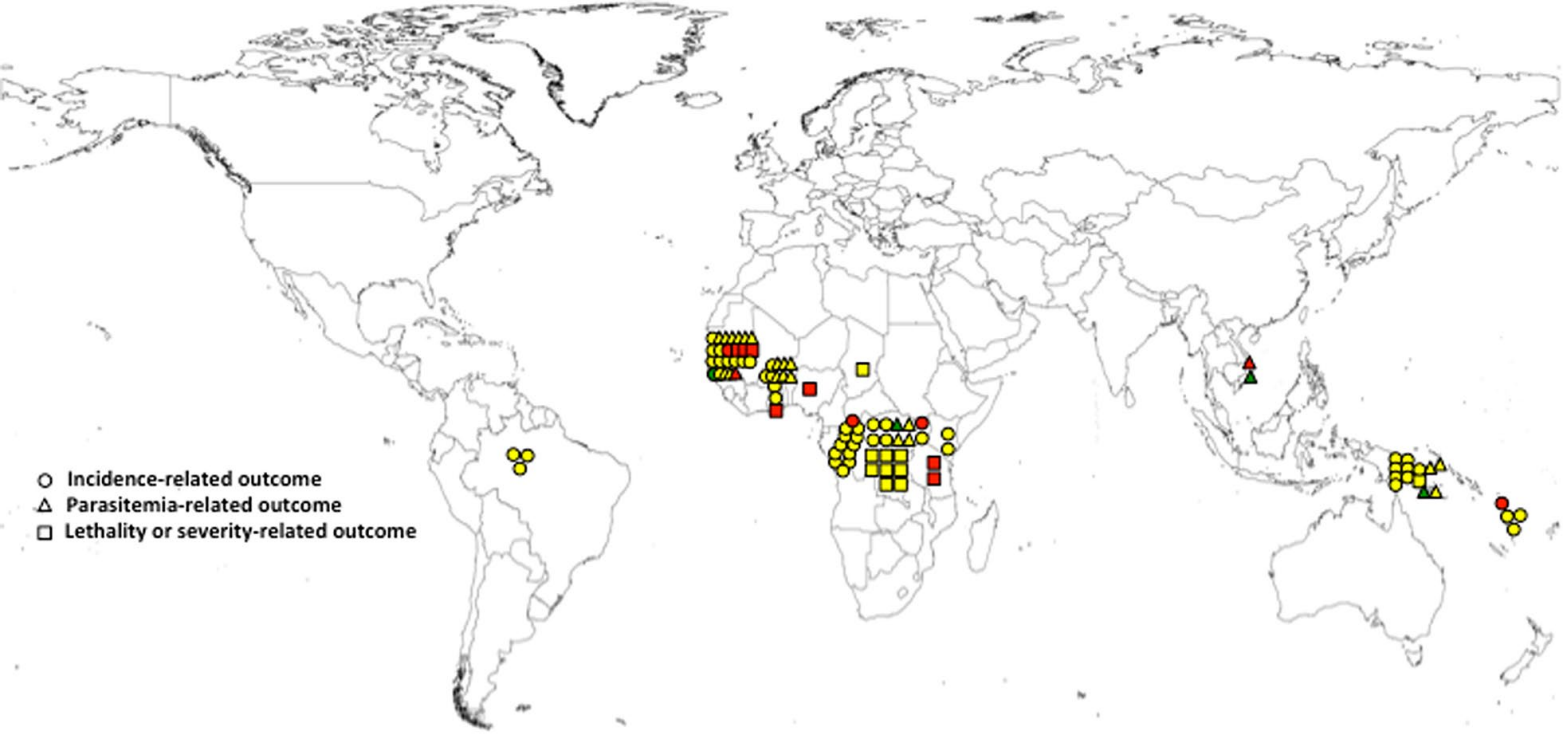
Distribution by country of the assessments from the studies on malnutrition as exposure to malaria retrieved in this systematic review, including the type of outcome and association found. *Colors* indicate the type of association: *Red* risk, *Yellow* absence of association, *Green* protection

The most assessed anthropometric measures were W/A, with 25 evaluations from 14 studies, and H/A, with 27 evaluations from 11 studies. The W/H measure was obtained in 24 evaluations from 10 studies. The measure AC/A was obtained in four evaluations from two studies. All the other measures (AC, weight increment, W/A increment, H/A increment, W/A increment, AC/A increment and a combined index from H/A + L/A + W/H + W/A measures) were obtained in two evaluations from one study each. Thus, a total of 92 different anthropometric measures were obtained from the 20 studies, being 14 using National Center Health Statistics 1977 [25], four the World Health Organization 2006 (WHO 2006) [26] and one the WHO 2007 [27] as standards. One study used weight increment (Fig. 2).

#### Malaria incidence

The impact of malnutrition on malaria incidence was assessed in 50 statistical comparisons from 12 studies, on parasite density in 25 comparisons from six studies and on mortality in 17 comparisons from seven studies (Table 2). Of the 50 comparisons between malnutrition and malaria incidence, 37 evaluated clinical malaria in general, four evaluated specifically asymptomatic malaria, four evaluated *Plasmodium falciparum* malaria, two evaluated *Plasmodium vivax* malaria, two evaluated *Plasmodium malariae* malaria and one evaluated specifically non-severe malaria. Most of the evaluations failed to show any association between malnutrition and malaria incidence; 45/50 (90 %). However, three evaluations showed a risk association between malnutrition and malaria incidence in children: (1) underweight on *P. vivax* incidence in children above 10 years with undisclosed follow-up time [30]; (2) AC/A on *P. falciparum* incidence in children under 9 months years with 3 months of follow-up [32]; (3) Stunting on *P. falciparum* incidence in children between 0 and 5 years with 20 weeks of follow-up [33]. Two evaluations showed a protective association: (1) Stunting on malaria incidence in children between 12 and 70 months with 25 weeks of follow-up [17]; (2) stunting on *P. vivax* incidence in children up to 14 years with 1 year of follow-up [38].

#### Parasite density

The 25 assessments of the relationship between malnutrition and malaria parasite density were highly heterogeneous: ten evaluated high parasite density (≥5000 parasites/mL), three evaluated high parasite density as ≥10,000 parasites/mL, three evaluated high parasite density as ≥5000 parasites/mL in asymptomatic malaria, three evaluated high parasite density as ≥300 parasites/mL, three evaluated high parasite density as ≥5000 parasites/mL specifically in *P. falciparum* malaria, three evaluated high parasite density as ≥10,000 parasites/mL specifically in *P. falciparum* malaria, one evaluated time to peak parasitaemia and one evaluated time to peak gametocytaemia.

Although the majority of studies showed no association between malnutrition and parasite density 20/25 (80 %); two evaluations showed a risk association: (1) Weight increments on time to peak *P.falciparum* parasite density in children of undisclosed age and 28 days of follow-up [28]; (2) Stunting on *P.falciparum* parasite density above 300/µL in children above 5 years with 25 weeks of follow-up [17]; while three comparisons showed a protective association: (1) weight increments on time to peak *P.falciparum* gametocytaemia in children of undisclosed age and 28 days of follow-up [28]; (2) Stunting on *P.falciparum* parasite density of above 5000/µL in children above 5 years with undisclosed follow-up time [16] and (3) Stunting on *P.falciparum* parasite density of above 5000/µL in children between 0 and 5 years with 1 year of follow-up [15].

#### Mortality and severity

Of the 17 comparisons between malnutrition and mortality, three evaluated malaria, two focused on severe malaria, one in cerebral malaria, and one evaluated three forms: malaria, cerebral and malarial anaemia. There were 10/17 (52.8 %) evaluations with no association between malnutrition and mortality. Seven assessments showed a risk association between malnutrition and mortality in children: (1) underweight on falciparum clinical, cerebral and malarial anaemia in children between 0 and 5 years with undisclosed follow-up time [2]; (2) underweight on falciparum mortality in children 1–7 months and between 8 months and 4 years with 1 year of follow-up [31]; (3) underweight on falciparum death or recover with neurological deficit on cerebral malaria in children between 1 and 5 years with undisclosed follow-up time [7]; (4) wasting on falciparum severe malaria in children above 5 years with undisclosed follow-up time [35].

### Malaria as exposure for malnutrition

**Fig. 3:**
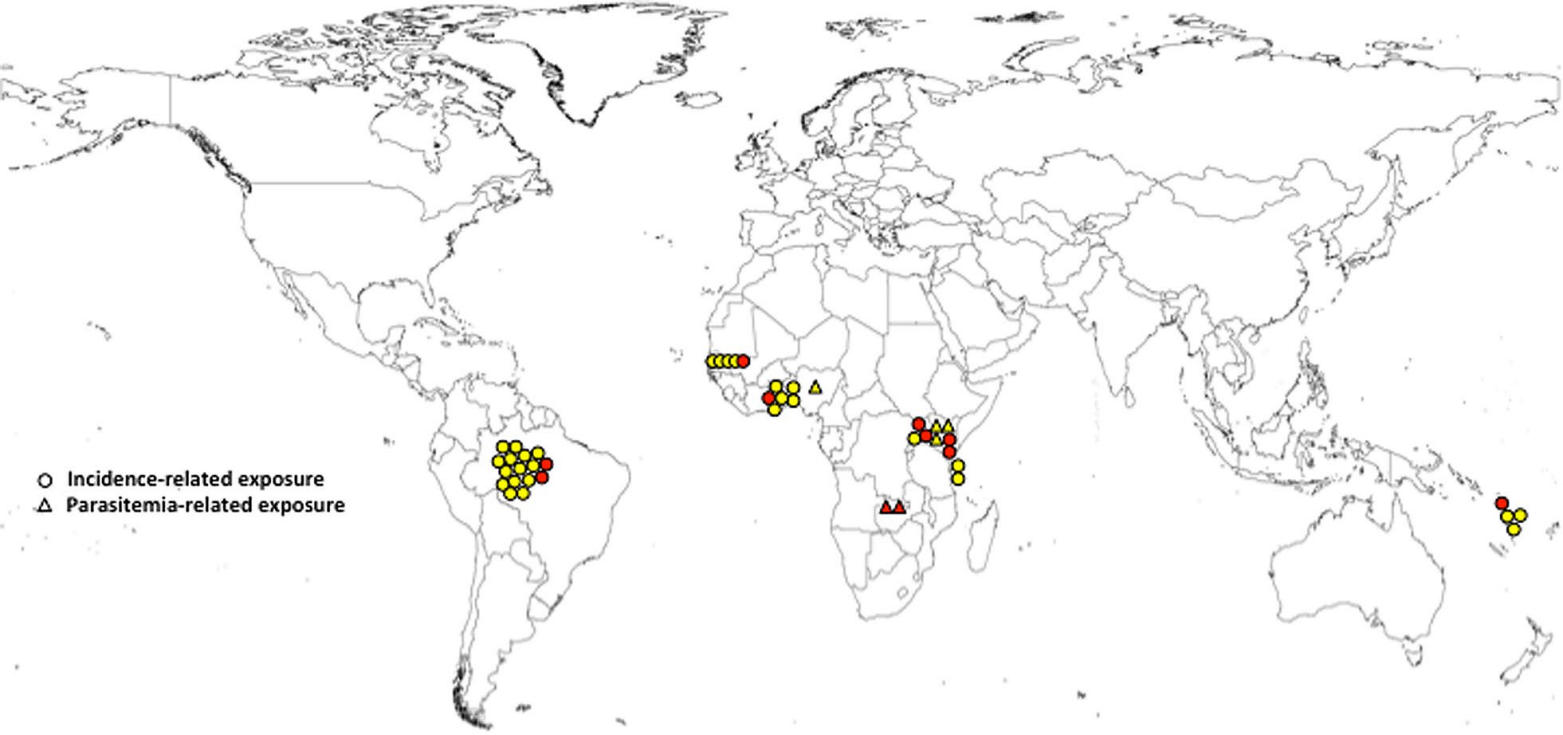
Distribution by country of the assessments from the studies on malaria as exposure to malnutrition retrieved in this systematic review, including the type of exposure and association found. *Colors* indicate the type of association: *Red* risk, *Yellow* absence of association, *Green* protection

Thirteen studies assessed the impact of malaria in nutrition, of them 10 assessed the role of malaria incidence (yes/no) while the remaining three used parasite density as the exposure of interest. The impact of malnutrition on malaria incidence was measured in 39 assessments from 10 studies and on parasite density in six assessments from three studies (Table 3; Fig. 3).

#### Anthropometric parameters

The most frequently assessed anthropometric measures were H/A, or L/A with 15 evaluations from nine studies, and W/H or W/L with eight evaluations from six studies. The W/A measure was obtained in seven evaluations from six studies. The measure BMI/A was obtained in five evaluations from two studies and so were height increments. The measure of BMI was evaluated two times in one study and the weight increment and fall in weight were obtained once. Thus, a total of 45 different anthropometric measures were obtained from the 13 studies, being four using NCHS, 1977 [25], five the WHO 2006 [26], one using CDC/NCHS (2000) [48] and one the WHO 2007 [27] as standards. Two studies used weight and/or height change.

Most such evaluations did not show an association between malaria and malnutrition 31/39 (79.4 %). Eight showed a risk association between malaria and subsequent malnutrition in children: (1) incidence of unspecified malaria on increment in weight in children between 0 and 5 years with 1 year of follow-up [40]; (2) Incidence of vivax malaria on W/H in children above 5 years with undisclosed follow-up time [30]; (3) incidence of falciparum malaria on H/A in children between 28 and 60 months with 1 year and 5 months of follow-up [36]; (4) incidence of unspecified malaria on H/A or L/A in children between 3 months to 2 years with 1 year and 9 months of follow-up [37]; (5) incidence of falciparum malaria on L/A and W/A in children from birth to 1 year with 1 year of follow-up [46]; (6) incidence of vivax/falciparum malaria on increment in height in children 5–10 years and 0–14 years with 1 year of follow-up [39].

Two out of six (20 %) assessments showed a positive association between parasite density and malnutrition in children: (1) mean falciparum/mixed malaria parasite density on H/A in children 6–9 months and 14–20 months with 1 year and 9 months of follow-up [41]; (2) mean falciparum parasite density on BMI in children above 5 years with undisclosed follow-up time [42].

### Methodological quality of studies

Of the 20 studies with malarial outcomes, fifteen had high and five had average quality according to the questionnaire of 12 items, with an average score of 80.5 %. The main methodological problem in these studies was not describing the main outcome measures (diagnostic technique for malaria and nutritional status). Of the fifteen high-quality studies, eight presented a risk association. Of the five medium quality, one found a risk association, totaling nine studies that found malnutrition as a risk factor for malaria (four in incidence, four mortality, and one in parasite density) (Tables 4, 5).

**Table 4:** Methodological quality of the longitudinal studies on anthropometric measures as exposures with malaria-related outcomes

Identification	Question												
	1	2	3	4	5	6	7	8	9	10	11	12	Score (%)
Pazzaglia et al. [28]	Y	Y	N	Y	N	Y	N	Y	N	N	Y	Y	58.3
Snow et al. [29]	Y	N	N	Y	Y	N	N	Y	Y	N	–	Y	54.5
Van den Broeck et al. [21]	Y	N	N	N	Y	N	Y	Y	Y	Y	N	Y	58.3
Genton et al. [22]	Y	Y	Y	Y	Y	Y	Y	Y	Y	N	–	Y	90.9
Olumese et al. [7]	Y	Y	N	Y	Y	Y	N	Y	Y	N	Y	Y	75.0
Renaudin et al. [20]	Y	N	Y	Y	N	N	Y	Y	Y	N	N	Y	58.3
Williams et al. [30]	Y	Y	Y	Y	Y	N	N	Y	Y	Y	–	Y	82.8
Man et al. [2]	Y	Y	N	Y	Y	N	N	Y	Y	N	N	Y	58.3
Genton et al. [16]	Y	Y	Y	Y	Y	Y	N	Y	Y	Y	Y	Y	91.6
Schellenberg et al. [31]	Y	Y	Y	Y	Y	N	Y	Y	Y	Y	Y	Y	91.6
Tonglet et al. [32]	Y	N	N	Y	Y	Y	Y	Y	Y	N	Y	Y	75.0
Deen et al. [33]	Y	N	Y	Y	Y	Y	Y	Y	Y	Y	Y	Y	91.6
Müller et al. [34]	Y	Y	Y	Y	Y	Y	N	Y	Y	Y	Y	Y	91.6
Mockenhaupt et al. [35]	Y	Y	Y	Y	Y	Y	Y	Y	Y	N	Y	Y	91.6
Nyakeriga et al. [36]	Y	Y	Y	Y	Y	Y	N	Y	Y	Y	Y	Y	91.6
Danquah et al. [37]	Y	N	N	Y	Y	Y	Y	Y	Y	N	Y	Y	75.0
Fillol et al. [17]	Y	Y	Y	Y	Y	Y	Y	Y	Y	Y	Y	Y	100.0
Arinaitwe et al. [38]	Y	N	Y	Y	Y	Y	Y	Y	Y	Y	Y	Y	91.6
Mitangala et al. [15]	Y	Y	Y	Y	Y	Y	Y	Y	Y	Y	Y	Y	100.0
Alexandre et al. [39]	Y	Y	Y	Y	N	Y	Y	Y	Y	Y	N	Y	83.3

(–) not informed

**Table 5:** Methodological quality of the longitudinal studies with malaria-related exposures and nutritional-related outcomes

Identification	**Question**												
	1	2	3	4	5	6	7	8	9	10	11	12	Score (%)
Rowland et al. [40]	Y	N	N	Y	Y	N	N	Y	Y	Y	-	Y	63.6
Williams et al. [30]	Y	Y	Y	Y	Y	N	N	Y	Y	Y	-	Y	81.8
Hautvast et al. [41]	Y	Y	Y	Y	Y	Y	Y	Y	Y	Y	Y	Y	100.0
Deen et al. [33]	Y	N	Y	Y	Y	Y	Y	Y	Y	Y	Y	Y	91.6
Friedman et al. [42]	Y	N	Y	Y	Y	Y	Y	Y	Y	Y	Y	Y	91.6
Nyakeriga et al. [36]	Y	Y	Y	Y	Y	Y	N	Y	Y	Y	Y	Y	91.6
Sowumi et al. [43]	Y	Y	Y	Y	Y	Y	N	Y	Y	Y	Y	Y	91.6
Danquah et al. [37]	Y	N	N	Y	Y	Y	Y	Y	Y	N	Y	Y	75.0
Kang et al. [44]	Y	N	Y	Y	Y	N	N	Y	Y	Y	Y	Y	75.0
Olney et al. [45]	Y	N	N	Y	N	N	Y	Y	Y	Y	Y	Y	66.6
Muhangi et al. [46]	Y	Y	Y	Y	Y	Y	Y	Y	Y	Y	-	Y	90.9
Padonou et al. [47]	Y	Y	Y	Y	Y	Y	Y	Y	Y	Y	Y	Y	100.0
Alexandre et al. [39]	Y	Y	Y	Y	N	Y	Y	Y	Y	Y	N	Y	83.3

(-) not informed

Of the 13 studies assessing nutritional status as the outcome, eleven had high and two medium quality according to the questionnaire of 12 items, with a mean score of 84.8 %. The main methodological problem in these studies was not clearly describing the exposure and the outcome (malaria species or anthropometric indicators with their anthropometric references). Of the eleven high quality studies, six had positive associations. Of the two medium quality, one found a positive association, totaling seven studies that found malaria as risk factor for malnutrition (5 using malaria incidence and 2 using parasite density).

## Discussion

Previous studies on the association of malaria and malnutrition delivered inconsistent results. These conflicting results could be explained by different levels of confounding and to considerable methodological dissimilarities. Randomized controlled trials are impractical and previous observational studies have not fully controlled for potential confounding including nutritional deficiencies, breastfeeding habits, other infectious diseases and socioeconomic status [46]. For the purpose of this systematic review, we restricted the nutritional assessment metrics to the above listed anthropometric measures as these have shown to be strong, reproducible indicators [49]. A significant heterogeneity in the assessed studies was observed in terms of sample size, follow-up time, exposure and outcome definitions, thus impeding a metanalysis, but in general the methodological quality of the retrieved studies was good enough to generate important conclusions. Here, results from longitudinal studies were emphasized, thus allowing temporal definitions, but quality of anthropometric measurements and their comparability across studies may constitute limitations.

The majority of studies showed no association between malnutrition and subsequent malaria incidence or parasite density. One study suggested a protective effect against malaria for malnutrition diagnosed by W/A [17]. In contrast, other studies have found an increased risk of malaria among stunted and underweight children [30, 32, 33, 38]. Earlier studies showed the presence of malaria in famine victims within few days of re-feeding and suggested that feeding provided essential nutrients for sequestered parasites leading to recrudescent infection [50, 51]. Whether other nutritional parameters are involved in the pathophysiology of nutrition and malaria is still controversial. The role of vitamin A, iron, zinc and other micronutrients in malaria is still a topic of debate [52]. In monkeys and mice, a low-protein diet was associated to a lower parasitaemia [53–55]. Most works found greater effects in younger age groups [17, 32, 36].

Measuring the association between measures of malnutrition and the risk of malaria is complex and is hampered by the multiplicity of metrics used for malnutrition. Stunting is generally considered an indicator of chronic malnutrition, wasting generally reflects a more recent and severe process, and being underweight is likely the result of both factors [38]. The results presented in this review support that malnutrition has not a great impact on malaria incidence and parasitaemia, although in a few epidemiological scenarios stunting, underweight and decreased increment on weight over time were measures of chronic malnutrition associated with an increased risk of malaria or high parasite density. Children who are underweight likely have increased susceptibility to malaria, most notably through a reduction in the function of the immune system [56]. An undernourished child is unable to mount an appropriate immune response to the malaria parasite due to reduction in T lymphocytes, impairment of antibody formation, decreased complement formation and atrophy of lymphoid tissues [1, 17]. Monkeys maintained on a low-protein diet were unable to clear the infection, resulting in multiple recrudescences [53]. Immune responses were also suppressed and parasitaemia appeared earlier and lasted longer in non-human primates [57].

The impact of malnutrition was noted mostly in malaria mortality and severity. Death or recovery with a neurological deficit on cerebral malaria was significantly associated with malnutrition in Nigerian children [7]. Underweight was identified as a risk factor for mortality, cerebral malaria and malarial anaemia in Gambian children [2] and with mortality in Tanzanian children, mostly in younger age groups [31]. Wasting was associated to mortality in children above 5 years in Ghana [35]. Some series of cases showed that cerebral malaria was more prevalent in well-nourished children than in children with severe malnutrition including kwashiorkor or marasmus [12, 13, 58]. In this study, cohort studies that evaluated mortality and other adverse malaria outcomes show risk of severe malnutrition [2, 7, 31, 35]. As shown previously, anthropometric measurements are consistently related to the risk of outcomes such as mortality in community-based studies from Asia and Africa [59]. In mice, it was noted an increased lethality in severely malnourished animals [60]. Although information on sensitive treatment surveillance was generally absent in the studies, it use some locations would be biased toward detecting differences in the onset of a malaria episode and not necessarily the subsequent severity. In malnutrition as exposure to malaria morbidity, studies that found protective associations had at least 1 year of follow-up whereas studies with risk associations only followed for 6 months or less. Most of the associations were found for height/age index, a marker of stunting or chronic malnutrition.

Anthropometric interaction of malaria and nutrition is confounded by micronutrient status, which also impacts on malaria severity [61], but unfortunately this potential bias was approached only in one study. The inability of anthopometry to distinguish the effect of specific nutrient deficiencies that affect growth in children is another limitation of this method [62]. Only five studies controlled for malaria treatment exposure [17, 22, 38, 44, 47] and four controlled for vector control measures [15, 16, 34, 47]. Prevention of malaria by intermittent preventive treatment was found to improve weight status in children [42, 63]. If malaria-specific interventions are responsible for this accelerated linear growth rate, the most likely explanation is that early treatment of clinical malaria prevented prolonged carriage of *P. falciparum* and its associated growth-depressing immune response [63]. Thus, a bias can be present in studies without this control. Different anthropometric assessment methods from studies with small sample sizes limited the comparability between findings retrieved in this study. It is known that WHO standards provide a better tool to monitor the rapid and changing rate of growth in early infancy [64]. Results from the retrieved studies were controlled for socioeconomic factors in a few cases, increasing the possibility of confounding [49].

*Plasmodium vivax* infection has been associated with severe malaria and death [65], although the risk of severe vivax malaria and case fatality rates are not well defined [66]. Co-morbidities are considered important contributors to severe complications of *P. vivax* infection. In particular, concomitant malnutrition is suspected to increase the risk of severe vivax disease, but this is not well understood. The demographic risk of severe vivax malaria in regions of relatively high endemicity is skewed towards early infancy (a stage when severe anaemia is a major cause of morbidity). A clearer picture of severe vivax malaria is emerging, but further studies are required to refine existing knowledge of the spectrum of syndromes and its association with malnutrition [39].

The relative insensitivity of anthropometry to detect changes in nutritional status over short periods of time limited conclusions from studies of short follow-ups. One speculates that hypercatabolism and inflammatory status induced by malaria may have an effect on nutritional well-being. TNF is a known mediator of anorexia and cachexia seen in many human disease states and is elevated during acute malaria [67–69]. In this systematic review, most of the studies showed no association between malaria incidence and malnutrition. As most anthropometric measures used gauged chronic malnutrition, one could speculate that if malaria has an effect on nutritional status, it would have be assessed with metrics targeting acute malnutrition. In areas where malaria is hyper-endemic, repeated infections throughout life may contribute to the burden of malnutrition, specially in children [36, 37, 40, 46]. No risk associations were found for malaria as exposure to malnutrition in studies with less than 1 year of follow-up.

Interestingly, in Vanuatu and in the Brazilian Amazon, where *P. vivax* contributes with a great percentage of the malaria cases, malaria has been associated with malnutrition in children [30, 39]. In these areas there is a general consensus stating that *P. vivax* would not be associated with malnutrition due to the misleading information that vivax malaria is benign [66]. However, this parasite species is able to develop dormant stages (hypnozoites) in the liver leading to frequent relapses, even months after the primary infection [70]. One especulates that this chronic relapsing nature of *P. vivax* may lead to a degree of chronic inflammation. Whether relapsing malaria significantly impairs nutritional status in *P. vivax* affected areas warrants further research.

## Conclusion

In conclusion, this systematic review found that the majority of studies assessing malaria and malnutrition were carried out in African *P. falciparum* endemic areas, with a significant study heterogeneity in terms of sample size, follow-up time and exposure and outcome definitions. Considering malnutrition as exposure, the results presented in this review support that malnutrition has not a great impact on malaria incidence and parasitaemia, but a greater negative impact of malnutrition was noted in malaria mortality and severity. A scarcity of prospective studies have been carried out aiming to establish the relationship between nutritional status and severity of vivax infection, this being a prioritary research topic. Most of the assessed studies showed no association between malaria incidence and malnutrition in areas of *P. falciparum* predominance, although the anthropometric parameters used were aimed mostly at chronic malnutrition, possibly suggesting that the impact of malaria on nutritional status may be of little significance in the long term. Interestingly, in areas where *P. vivax* contributes with a great percentage of the cases, malaria was associated with risk for malnutrition in children. A discussion among experts in the field is needed to standardize the observational studies considering external validity in order to allow for more accurate conclusions.
